# New views on the hypothesis of respiratory cancer risk from soluble nickel exposure; and reconsideration of this risk's historical sources in nickel refineries

**DOI:** 10.1186/1745-6673-4-23

**Published:** 2009-08-23

**Authors:** James G Heller, Philip G Thornhill, Bruce R Conard

**Affiliations:** 1 James G. Heller Consulting Inc., 1 Berney Crescent, Toronto ON, M4G 3G4, Canada; 2 Dalla Lana School of Public Health, University of Toronto, 6th Floor, Health Sciences Building, 155 College Street, Toronto ON, M5T 3M7, Canada; 3 Metallurgical Research, Falconbridge Ltd, Toronto ON, Canada; 4 Environmental and Health Sciences, Inco Ltd, Toronto, ON, Canada; 5 BR Conard Consulting, Inc., 153 Balsam Drive, Oakville ON, L6J 3X4, Canada

## Abstract

**Introduction:**

While epidemiological methods have grown in sophistication during the 20^th ^century, their application in historical occupational (and environmental) health research has also led to a corresponding growth in uncertainty in the validity and reliability of the attribution of risk in the resulting studies, particularly where study periods extend back in time to the immediate postwar era (1945–70) when exposure measurements were sporadic, unsystematically collected and primitive in technique; and, more so, to the pre-WWII era (when exposure data were essentially non-existent). These uncertainties propagate with animal studies that are designed to confirm the carcinogenicity by inhalation exposure of a chemical putatively responsible for historical workplace cancers since exact exposure conditions were never well characterized. In this report, we present a weight of scientific evidence examination of the human and toxicological evidence to show that soluble nickel is not carcinogenic; and, furthermore, that the carcinogenic potencies previously assigned by regulators to sulphidic and oxidic nickel compounds for the purposes of developing occupational exposure limits have likely been overestimated.

**Methods:**

Published, file and archival evidence covering the pertinent epidemiology, biostatistics, confounding factors, toxicology, industrial hygiene and exposure factors, and other risky exposures were examined to evaluate the soluble nickel carcinogenicity hypothesis; and the likely contribution of a competing workplace carcinogen (arsenic) on sulphidic and oxidic nickel risk estimates.

**Findings:**

Sharp contrasts in available land area and topography, and consequent intensity of production and refinery process layouts, likely account for differences in nickel species exposures in the Kristiansand (KNR) and Port Colborne (PCNR) refineries. These differences indicate mixed sulphidic and oxidic nickel and arsenic exposures in KNR's historical electrolysis department that were previously overlooked in favour of only soluble nickel exposure; and the absence of comparable insoluble nickel exposures in PCNR's tankhouse, a finding that is consistent with the absence of respiratory cancer risk there. The most recent KNR evidence linking soluble nickel with lung cancer risk arose in a reconfiguration of KNR's historical exposures. But the resulting job exposure matrix lacks an objective, protocol-driven rationale that could provide a valid and reliable basis for analyzing the relationship of KNR lung cancer risk with any nickel species. Evidence of significant arsenic exposure during the processing step in the Clydach refinery's hydrometallurgy department in the 1902–1934 time period likely accounts for most of the elevated respiratory cancer risk observed at that time. An understanding of the mechanism for nickel carcinogenicity remains an elusive goal of toxicological research; as does its capacity to confirm the human health evidence on this subject with animal studies.

**Concluding remarks:**

Epidemiological methods have failed to accurately identify the source(s) of observed lung cancer risk in at least one nickel refinery (KNR). This failure, together with the negative long-term animal inhalation studies on soluble nickel and other toxicological evidence, strongly suggest that the designation of soluble nickel as carcinogenic should be reconsidered, and that the true causes of historical lung cancer risk at certain nickel refineries lie in other exposures, including insoluble nickel compounds, arsenic, sulphuric acid mists and smoking.

## Introduction

While epidemiological methods have grown in sophistication during the 20^th ^century, their application in historical occupational (and environmental) health research has also led to a corresponding growth in uncertainty in the validity and reliability of the attribution of risk in the resulting studies, particularly where study periods extend back in time to the immediate postwar era (1945–70) when exposure measurements were sporadic, unsystematically collected and primitive in technique; and, more so, to the pre-WWII era (when exposure data were essentially non-existent). These uncertainties propagate with animal studies that are designed to confirm the carcinogenicity by inhalation exposure of a chemical putatively responsible for historical workplace cancers since the exact historical exposure conditions were never well characterized. In this report, we present human and toxicological evidence to show that soluble nickel is not carcinogenic; and, furthermore, that the carcinogenic potencies previously assigned by regulators to sulphidic and oxidic nickel compounds for the purpose of developing occupational exposure limits have likely been overestimated. [Note to the reader: Nickel-containing substances can be grouped into five main classes based on their physicochemical characteristics: nickel carbonyl (gas), metallic nickel (e.g., elemental nickel, nickel-containing alloys), oxidic nickel (e.g., nickel oxides, hydroxides, silicates, carbonates, complex nickel oxides), sulphidic nickel (e.g., nickel sulphide, nickel subsulphide) and water soluble nickel compounds (e.g., nickel sulphate hexahydrate, nickel chloride hexahydrate). Exposures during nickel refining may contain several of these nickel species depending on the type of process used.]

Support for the soluble nickel carcinogenicity hypothesis was found in the epidemiological findings at two refineries, involving high exposure to soluble nickel, i.e. nickel sulphate hexahydrate (1–5 mg/m^3^), of workers in the electrolysis department at the Kristiansand Nikkelrafferingsverk refinery (KNR) in Norway [1-8] and the hydrometallurgy department at Clydach Wales [3]. These findings led the International Committee on Nickel Carcinogenesis in Man (ICNCM) to conclude in 1990 that *'soluble nickel exposure increased the risk of these cancers *[lung and nasal] *and that it may enhance risks associated with exposure to less soluble forms of nickel *[i.e. sulphidic and oxidic nickel]' ([3].pp74). The ICNCM exercised caution and prudence in this conclusion despite available contradictory epidemiological evidence from a nickel refinery study in Port Colborne Ontario (PCNR) that found no increased risk of lung cancer among its electrolysis workers who also had soluble nickel exposures comparable to those in the corresponding KNR department [9,10]. Both refineries (KNR and PCNR) used the Hybinette electrolytic refining process [11,12] and, although PCNR electrolysis workers had somewhat less exposure to airborne soluble nickel than KNR workers, differences were likely due in part to the classification of nickel carbonate as insoluble at PCNR and as soluble at KNR. KNR electrolysis workers reportedly experienced higher levels of insoluble nickel exposures than did PCNR workers, especially before 1967 ([3].pp20).

The present paper focuses primarily on published KNR human health studies for two reasons: (1) because KNR studies still show lingering respiratory cancer risk after 30 years of epidemiological studies, which, if true, must raise serious occupational and public health concerns for Norwegian health authorities; and (2) because it remains in current production, KNR's evidence provides the gravitas of evidentiary support for soluble nickel's carcinogenicity. The Clydach refinery era of epidemiological interest in this respect extended from 1902 to 1937 after which time the throughput on Clydach's copper extraction (copper plant) and nickel sulphate refining (hydrometallurgy) departments had been considerably reduced. By 1948, the copper leaching step on calcines and the nickel sulphate recycle were eliminated, ending the nickel-copper oxide dust and nickel sulphate spray and mist hazards in the copper plant ([3].pp15–16).

In its investigations, the ICNCM reported that no measurements of actual nickel concentrations, let alone nickel species, existed in the workplaces of any nickel plant operations before 1950 ([3].pp11). Very few measurements were available before the early 1970s for the KNR refinery ([3].pp15–16), and likely for the Welsh refinery as well. In the absence of real exposure data, therefore, the range and percentage of total airborne nickel (and of nickel species) were estimated on the basis of process knowledge, subjective impressions of relative dustiness, and a few measurements ([3].pp12–13). KNR historical exposure data were similarly based on the subjective judgements of retired personnel with the distribution of nickel species in airborne dust assumed to be the same as that in the bulk feeds and materials handled ([3].pp15–16). In their Clydach risk-exposure modeling study, Easton *et al*. rightly acknowledged the uncertainties in their nickel species-specific cancer risk models, which they found to be highly sensitive to small shifts in the historical values imputed to insoluble and soluble nickel exposures [13].

Focusing the human health studies exclusively on nickel without considering exposures from nuisance carcinogens in the mined nickel ore and production steps has also meant that few recorded measurements of these contaminants (viz. arsenic, sulphuric acid mists) are available today to estimate their possible contribution to observed carcinogenic risk. The established human health evidence on nickel has necessarily influenced the interpretation of nickel toxicology studies as well. In this paper, we will demonstrate that epidemiological studies have not proven that soluble nickel is carcinogenic. Indeed, this shift in the human health evidence must change the interpretation of soluble nickel's toxicology, and raise questions for regulatory toxicologists to consider concerning possible overestimation of the carcinogenic potencies previously assigned to sulphidic and oxidic nickel.

## Methods

We examined in detail all published reports of occupational cancer in nickel operations around the world with environmental exposures to soluble nickel, including refineries at Kristiansand Norway [1-8], Clydach Wales [3,14-21], Port Colborne Ontario [9,10], Thompson Manitoba [F1: Roberts RS, Jadon N and Julian JA: A mortality study of the INCO Thompson workforce. McMaster University, 1991. Available from the authors], and Harjavalta Finland [22,23]; and a British nickel-plating company [24]. We also obtained file and archival information from the KNR and PCNR environmental departments. Our examination included: historical production processes, environment and hygiene issues at both refineries; personal files, including a detailed report, filed with the ICNCM, of KNR's building development, process steps and exposure patterns over the 1910–1986 period [F2: Thornhill PG: The Kristiansand Refinery: A description of the Hybinette Process as practised 1910 to 1978. Falconbridge Limited, Dec. 15, 1986. Available from Xstrata Nickel]; and the protocol for the construction of KNR's Job Exposure Matrix (JEM), originally developed for the ICNCM (1990) [3] study [F3: Protocol for Falconbridge Nikkelverk's Epidemiological Prospective Investigation (EPI) Study. February 21, 1986, 1^st ^protocol version. Also, Prospective Investigation Based on Employees from Falconbridge Nickel refinery, Kristiansand, Norway, Oslo/Kristiansand/Sudbury (Canada), October 1986, 2^nd ^protocol version. Available from Xstrata Nickel]. Environmental specialists at both refineries provided a range of materials, including datasets summarizing historical personal and area environmental measurements [F4: The Kristiansand Nikkelverk Refinery: History, Process Descriptions & Environmental Monitoring Data, 2005. Available from Xstrata Nickel] [F5: The Port Colborne Refinery: History, Process Descriptions & Environmental Monitoring Data, 2005. Available from Vale Inco Ltd.], the Glømme report that documented post-WWII KNR area sampling measurements through 1967 [F6: Glømme J: Arbeidshygieniske undersökelser over virkningen av irriterende gasser og forskjellige partikulæforurensingeer I arbeidsatmosfæren ïen norsk elektrokjemisk industri (Effect of irritating gases and different dust particles in the working atmosphere in a Norwegian electrochemical industry). 2 volumes. Kristiansands Nikkelraffineringsverk, Norway. August, 1967. Available from Xstrata Nickel], KNR environmental reports [F7: Wigstøl E and Andersen I: The Kristiansand Nickel Refinery: Production – Processes – Environment – Health. Falconbridge Nikkelverk A/S, 1985. Includes: Resmann F: Falconbridge Nikkelverk Aktieselskap. Memorandum to E. Wigstøl. Kristiansands Nikkelraffineringsverk, Norway. Dec. 23, 1977. Available from Xstrata Nickel], and a translation (from Norwegian) of a publication of KNR's history [25]. We reviewed a published study of historical environmental exposures in KNR's Roasting, Smelting and Calcining (RSC) department that was cited in support of the substantive changes to the original KNR JEM that resulted in the historical exposure dataset for all post-1998 KNR occupational health studies [26]. On the subject of arsenic exposures, we also examined published and file materials and anecdotal evidence on: (1) historical arsenic exposures in nickel refinery process operations arising from arsenic-rich nickel ores mined in the Sudbury basin [27] and putative associated risks [10,28,29]; (2) the presence of arsenic in KNR's purification section, which was connected to its Ni electrolysis department; and on (3) sulphuric acid contaminated with significant concentrations of arsenic that was used for copper extraction at Clydach during the critical time period of high respiratory cancer risk at this refinery (1902–1934) [14,27]. Finally, we examined the toxicological literature related to soluble nickel and related animal studies [30-43].

## Findings and discussion

### 1. The effects of topography and building architecture on the presence of insoluble nickel exposures in KNR's electrolysis department and their absence in PCNR's Ni tankhouse

**Figure 1:**
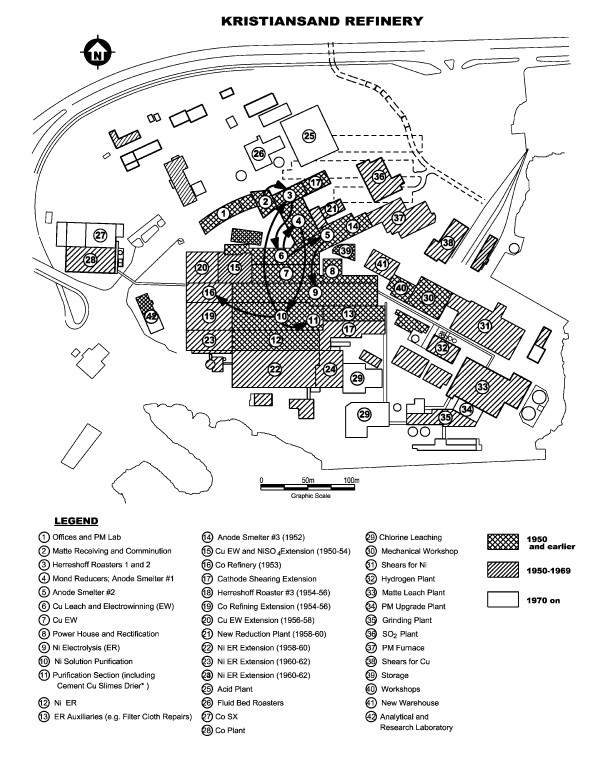
**Scale drawing of KNR showing building layouts and process flows by time period**. Note abutment and connection of key environments, including Ni ER [#9 and 12], and Ni and Cu purification [#10 and 11]. Sources: Thornhill (1986) [F2] & [F4].

**Figure 2:**
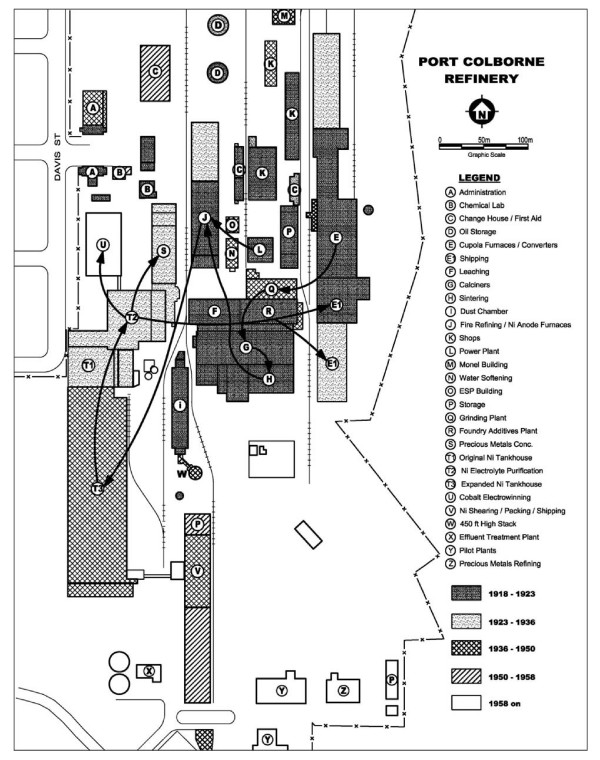
**Scale drawing of PCNR showing building layouts and process flows by time period**. Note physical separation of Ni tankhouse (electrolysis department) and leaching, calcining and sintering (LC&S) environments. Source: Vale Inco Ltd.

The KNR began operations in 1910 on a Norwegian fjord with a land base of 10 hectares of typical hilly terrain in order to access cheap power and transport by sea [25] (Figure 1). The PCNR began production in 1918 on 360 acres of a flat and uneventful former lake bed on the shores of Lake Erie, also to access cheap power and marine transport. PCNR's buildings and working areas occupied about 220 acres (89 hectares) of the property, almost 9 times the size of the comparable KNR foot print (Figure 2). Both plants employed the Hybinette electrolytic process, the final step in nickel refining and source of soluble and metallic nickel exposures in their respective electrolysis departments, which also carried trace level exposures to oxidic nickel but very low exposures to sulphidic nickel compounds. [Note to the reader: For complete accuracy, it is noted that a small portion of the PCNR tankhouse was devoted to electrolytic refining of sulphidic anodes starting in the mid-1950s until the Thompson refinery was commissioned in 1960. Exposure to nickel sulphides in the PCNR tankhouse would have been low and of relatively short duration.]

**Figure 3:**
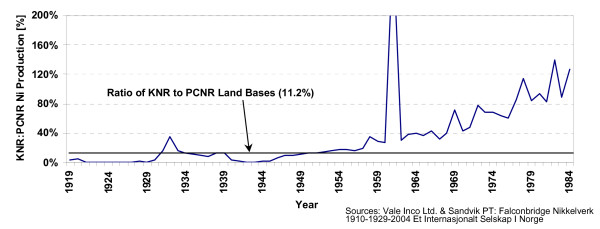
**Ratio of KNR to PCNR Nickel Production: 1919–1984**.

**Figure 4:**
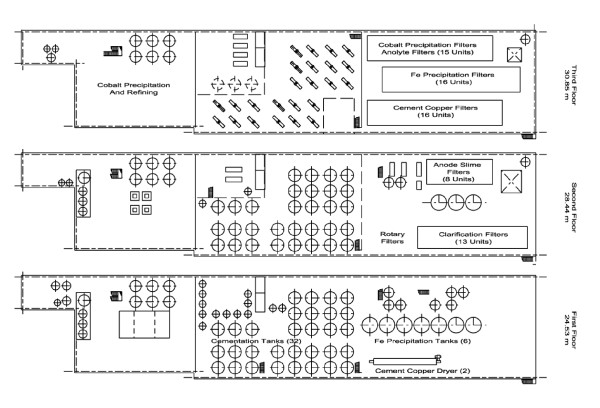
**Plan view of the three floors of KNR's Purification section**. Shows stacking and abutment where typical composition of arsenic in processed products before 1953 was 10.4% by weight. Source: Thornhill (1986) [F2].

**Figure 5:**
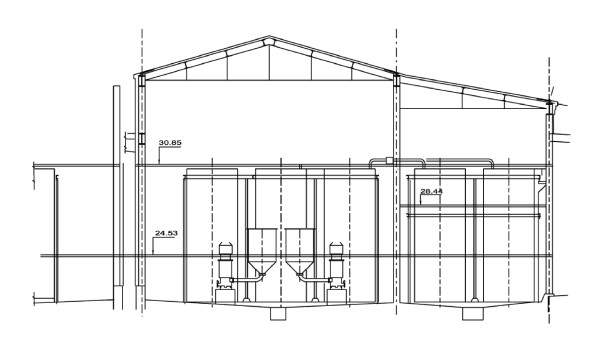
**Vertical section through row of KNR cementation tanks shown in Figure 4**. Source: Thornhill (1986) [F2].

KNR has a unique and eventful history that included partial destruction by fire and cessation of operation in 1918, followed by the refinery's repair and reopening only to face shutdown and bankruptcy during the twenties because of the sharp downturn in global nickel prices. Following its purchase by Falconbridge Nickel Mines Ltd in 1928, it was modernized and resumed operation in February 1930 [25]. The plant was occupied and operated by German forces from April 1940 to the cessation of hostilities in Europe in the summer of 1945. The following chart shows that, except for the shutdown in the twenties and the war period, KNR always operated more intensively (as measured in tons of nickel produced per year per hectare of land base) than PCNR (including 1961 when PCNR's production level fell by over 90%) (Figure 3). PCNR's flat topography and ample land base allowed physical separation of key buildings and horizontal process layouts. Unlike the PCNR facility, KNR's topography and foot print necessitated multi-storied building structures that either abutted each other or were connected by covered tramways linking successive process steps (Figure 4) (Figure 5) (Table 1). The schematics highlight building development, including the evolution of the Hybinette process refining steps over four time periods (i.e. 1910–29, 1930–49, 1950–69, 1970–78) [25], and support our contention of cross-contamination of KNR's electrolysis department environment by known carcinogens (sulphidic and oxidic nickel) originating within its RSC department. For example, Thornhill (1986) documented evidence, filed with the ICNCM, showing that KNR process workers received mixed dust exposures during such operations as the transfer of calcine by wheelbarrow until 1956 from KNR's roasting building to its electrolysis department [F2]. In 1954, about 150 tons per day of calcine were leached. Assuming a loading of 0.25 tons per trip, the workers would have been required to load and dump these barrows 600 times per day. Exposures to dust from these two operations would occur 1,200 times per day. After 1956, the transfer was by closed drag conveyor, which structure trapped fugitive dust that led to mixed exposures [F2].

**Table 1:** KNR Process Flow Descriptions in Figure 1

**Process Flows**	**Description**
(2) to (3)	Ground matte lifted to roasters @ 25 m elevation using bucket elevators (144 t/day)^a^
(3) to (3)	Cooled calcine to air classification in closed circuit regrind @ 35 m elevation (216 t/day)
(3) to (6)	Calcine to copper leach (205 t/day)
(6) to (5)	Residue fine fraction to anode smelting (97 t/day)
(5) to (9)^b^	Anodes to Ni electrorefining
(6) to (4)	Residue coarse fraction to Mond reducers before 1953 (hydrogen reduction after) (46 t/day)
(4) to (10)	Reduced Cu leach residue to copper cementation (38 t/day)
(10) to (3)	Cement Cu (17 t/day) and dried cement Cu slimes (23 t/day) to roasters^c^
(10) to/from (11)^d^	Cement Cu slimes to drying (40 t/day) before transfer to roasters^c^
(10) to (15)	Crude Cobaltic Hydroxide to Cobalt refinery

Sources: Thornhill (1986) [F2] and [F4]. ^a ^Ni substances handled daily in fine solids form (averages daily tonnages in 1958). ^b ^Includes deliver of anodes from building # 4 or 13 to # 11, 21, 22 or 23. ^c ^High As dust levels before 1953. ^d ^Building # 11 is a 3storey structure containing 32 Cu cementation tanks, extending through 1^st ^and 2^nd ^floors, and loaded from the 3^rd ^floor; 13 cement Cu filters (3^rd ^floor); 2 cement Cu driers (1^st ^floor); 15 Co precipitate filters (3^rd ^floor); 16 Fe precipitate filters (3^rd ^floor); 8 anode slime filters & 13 clarification filters (2^nd ^floor); and 6 Fe precipitation tanks (1^st ^floor). Workers in this section were classified as electrolysis workers. See Figures 4 and 5.

Differences in (1) land topography and footprints led to (2) differences in production intensity and to (3) differences in building architecture at the two refineries (including stacking, abutment and connection of key KNR department environments, and the isolation of PCNR's Ni tankhouse from its LC&S building and insoluble Ni carcinogenic exposures). Coupled with (4) KNR's disruptive production history, these factors all contributed to significant differences in each refinery's environmental hygiene history over the twentieth century and were likely responsible, in our opinion, for the presence of known insoluble nickel carcinogenic exposures (i.e. oxidic and sulphidic nickel) in KNR's historical electrolysis department and their comparative absence in the corresponding PCNR department. KNR researchers have criticized the PCNR study's mortality ascertainment methods, contending that it underestimated the carcinogenic risk of its electrolysis workers. Their critique is addressed fully by the analysis provided in Appendix 1 and accompanying tables (Table 14 and Table 15).

### 2. Exposure and worker misclassification issues in the published KNR epidemiology

KNR's epidemiology studies can be grouped for examination into three time periods distinguished by the methodology for assigning person years at risk (PYRs) to exposure categories defined by process department, job type, time period and nickel compound (Table 2).

**Table 2:** Characteristics of KNR epidemiological studies by treatment of worker exposure

**First Author (Year)**	**Follow up period**	**Year first employed**	**Number of workers**	**Cases of lung cancer**	**Qualifications for study entry^a^**
**I. Studies using rule based allocation of workers to process department**					
Pedersen (1973) [1]^b^	1953–71	1910–60	1,916	48	≥ 3 years employment; alive on Jan. 1, 1953
Magnus (1982) [2]^b^	1953–79	1916–65	2,247	82	≥ 3 years employment; alive on Jan. 1, 1953

**II. Studies using ICNCM Job Exposure Matrix developed by protocol**					
ICNCM (1990)[3]^b^	1953–84	1946–69	3,250	77	≥ 1 year employment; alive on Jan. 1, 1953
Andersen (1996) [4]^b^	1953–93	1916–40	379	203	≥ 3 years employment; alive on Jan. 1, 1953
		1946–83	4,385		≥ 1 year employment; alive on Jan. 1, 1953

**III. Studies using revised Job Exposure Matrix**					
Grimsrud (2002)[6]^c^	Dec '52-Aug '95	1910–94	5,389	227	≥ 1 year employment; alive on Jan. 1, 1953
Grimsrud (2003)[7]^b^	1953–2000	1910–89	5,297	267	≥ 1 year employment; alive on Jan. 1, 1953
Grimsrud (2005)[8]^c^	Dec '52-Aug '95	1910–94	5,389	227	≥ 1 year employment; alive on Jan. 1, 1953

^a ^A worker qualified on Jan. 1, 1953, or on the first succeeding date when he had the minimum qualifying employment. ^b ^Cohort study

^c ^Case control study

#### 2.1 KNR studies using rule based allocation of workers to process department

The earliest studies by Pedersen *et al*. (1973) [1] and Magnus *et al*. (1982) [2] adopted a rule based procedure to assign a worker's case (if he contracted cancer) and his PYRs to electrolysis, RSC or 'other specified' work processes, depending on which of these three categories he had spent the longest time even if it was less than half of his overall KNR employment experience (Table 3). The process classification rules in both studies made it impossible to distinguish respiratory cancer risk among the key roasting-smelting and electrolysis departments (Table 4); and even assigned nasal cancer risk implausibly to 'other specified processes' and administrative and service areas. Both studies found that cancer risk was elevated throughout the KNR refinery, an unlikely finding that signals the presence of misclassification problems. In retrospect, the Pedersen *et al*. [1] study was the first human health study to raise the hypothesis of soluble nickel's carcinogenicity in the scientific literature.

**Table 3:** Rules for classifying KNR workers by process and number of men by process in Pedersen *et al*. (1973) [1] and Magnus *et al*. (1982) [2]

	**# of men**		
		
**Categories of work**	**Pedersen (1973)**	**Magnus (1982)**	**Rules allocating workers to processes**
Roasting- smelting (R/S)	462	528	1) Cases and expected values (PYRs) for each process worker were classified to one of three processes (i.e. R/S, E or O) where he spent the longest time.
Electrolysis (E)	609	685	
Other specified processes (O)	299	356	2) If he only spent *some *time in process work, but *most *of his time in non-process work (e.g. labourers, plumbers, fitters, foremen, technicians, etc.), then his experience was classified to the process (i.e. R/S, E or O).
Other and unspecified work (U)	546	678	3) If he worked in unspecified process work *only*, then his experience was allocated to that process (i.e. U).
Total	1,916	2,247	

**Table 4:** Risk of respiratory cancer mortality in Pedersen *et al*. (1973) [1]; and respiratory cancer incidence in Magnus *et al*. (1982) [2]

	**Nasal cavities**		**Larynx**		**Lung**		**All respiratory organs**	
	
**Categories of work**	**Obs**	**SMR**	**Obs**	**SMR**	**Obs**	**SMR**	**Obs**	**SMR**
	**Pedersen *et al*. (1973)**							
	
Roasting-smelting	5	5000	4	1000	12	480	21	700
Electrolysis	6	3000	-	-	26	720	32	744
Other specified processes	1	1000	1	500	6	460	8	500
Administration, service and unspecified	2	2000	-	-	4	150	6	194
Total	14	2800	5	360	48	475	67	558

	**Magnus *et al*. (1982)**							
	
Roasting-smelting	8	4000	4	670	19	360	31	510
Electrolysis	8	2670	0	0	40	550	48	570
Other specified processes	2	2000	1	330	12	390	15	430
Administration, service and unspecified	3	1500	0	0	11	175	14	190
Total	21	2630	5	210	82	370	108	430

#### 2.2 KNR studies using ICNCM Job Exposure Matrix developed by protocol

The ICNCM provided the impetus for fresh research on nickel carcinogenicity at KNR. Research was governed by a protocol defining a rule based procedure, followed by a consensus committee of retired personnel, to review employment records and develop a JEM to assign species specific nickel exposures to every KNR worker [F3]. The protocol was developed by a team from Falconbridge KNR and Canada, the Norwegian Cancer Registry (NCR), and the Norwegian Institute of Occupational Health (NIOH) and chaired by one of us (Thornhill) who had specific responsibilities to gather and prepare data on species, specific historical exposures and their quantitative ranges, and to confirm results with KNR and NIOH officials. He recalled warning KNR researchers that the refinery's historical records could not support the *elevation *in individual worker exposure levels that would result from converting the original JEM's exposure categories from ordinal to continuous values (by averaging range boundaries).

The next table (Table 5) is drawn from the resulting KNR study published in the ICNCM (1990) report [3]. The estimates display the same problem identified in earlier studies, namely that lung cancer risk remained improbably elevated throughout the refinery including administrative and service department areas. This finding underlines the persistence of misclassification problems in KNR's epidemiology.

**Table 5:** Risk of lung cancer mortality among KNR workers with at least 15 years since first exposure by category of work, date of first exposure (for electrolysis & RSC departments) and duration of employment; ICNCM (1990) [3]

	**Duration of employment**								
	
**Category of Work**	**< 5 years**			**≥ 5 years**				**Total**	
	**Obs**	**SMR**		**Obs**	**SMR**		**Obs**	**SMR**	
Electrolysis:^1^									
First exposure: 1946–1955	10	318	*	16	482	***	26	402	***
First exposure: 1956–1969	1	152		3	448		4	300	
Electrolysis: Total	11	289	*	19	476	***	30	385	***
Roasting, Smelting and Calcining:^2^									
First exposure: 1946–1955	5	211		7	298		12	254	**
First exposure: 1956–1969	1	139		1	128		2	133	
RSC: Total	6	194		8	254	*	14	225	**
Other KNR Departments:^3^									
Low level exposure^4^	1	73		5	267		6	187	
Unexposed^4^	4	349		2	93		6	183	
Other departments: Total^5^	5	250		18	275	**	23	283	**
Refinery: Total^6^	22	247	**	45	334	***	67	299	***

^1 ^From Table forty three, ICNCM (1990) [3]. ^2 ^From Table forty four, ICNCM (1990) [3]. ^3 ^Labelled in previous KNR studies as 'other specified processes' and 'administrative, service and unspecified'. ^4 ^From Table forty five, ICNCM (1990) [3]. ^5 ^Calculated by equating time since first exposure in electrolysis or RSC departments with time since first employment in the refinery; and subtracting Electrolysis Total and RSC Total from Refinery Total. ^6 ^From Table forty one, ICNCM (1990) [3]. * p < 0.05. ** p < 0.01. *** p < 0.001.

These problems may be related to the presence of a part-time or seasonal subcohort. We discovered historical KNR employment data filed with the ICNCM that showed enormous annual turnovers in staff, averaging over 50% annually during the 1951–69 period (Table 6) [F2]. This finding supports the existence of a large part- time workforce of men entering and leaving the refinery every year (since it would have been impossible to train over 600 new job entrants annually). Part time workers may have circulated in more heavily exposed jobs and departments on the principle that seniority was the pathway to better jobs. Their employment records would be less likely to provide reliable documentation of their department and job histories, largely because they would have entered a labour pool where departmental foremen assigned jobs on the basis of daily requirements. Anecdotal reports suggest that these seasonal workers included local farmers and merchant seamen with their own acquired risk histories (pesticides for farmers, asbestos exposure for merchant seamen, etc.) [F8: Torjussen W and Andersen I: Cigarette smoking, nickel exposure and respiratory cancer. Kristiansand, Norway. 2005. Available from the authors]. Short-term workers are known to have poorer health, likely related to lower attained educational and income socio-economic status (SES) and heavier smoking behaviour (an ever-smoking prevalence of 82% was found in the historical KNR workforce [2]). No account of this workforce was provided in the published KNR studies, and failure to analyze its epidemiology separately may account for the misclassification issues.

**Table 6:** Turnover in Hourly-Rated KNR Employees: 1951–68*

**Year**	**As of Jan. 1**	**During Calendar Year**			**Percent Leaving^a^**
		
		**Total**	**Hired**	**1. Left**	
1951	795	1,250	455	419	51.5
1952	831	1,791	960	757	81.2
1953	1,034	1,951	917	841	78.5
1954	1,110	2,206	1,096	961	81.6
1955	1,245	2,165	920	902	71.9
1956	1,263	2,250	987	951	74.2
1957	1,299	2,111	812	878	69.4
1958	1,233	1,577	344	415	34.7
1959	1,162	1,440	278	317	27.7
1960	1,123	1,591	468	445	39.2
1961	1,146	1,733	587	547	46.9
1962	1,186	1,602	416	455	39.0
1963	1,147	1,319	172	304	28.1
1964	1,015	1,261	246	178	17.0
1965	1,083	1,684	601	612	56.8
1966	1,072	1,617	545	564	53.1
1967	1,053	1,447	394	463	45.5
1968	984	1,506	522	452	44.4
1969	1,054	1,809	755	708	67.2
Avg	1,097	1,701	604	588	53.0
SD	133	315	279	238	19.7

* Table X (revised) in Thornhill (1986) [F2]. ^a ^Number of men leaving expressed as a percentage of the average number of employees at start and end of year, except for the 1969 estimate, which is based on Jan. 1^st ^total. Avg: Average. SD: Standard deviation.

#### 2.3 KNR studies using revised Job Exposure Matrix

On the basis of environmental studies conducted in the nineties (discussed later), Grimsrud *et al*. (2000) revised the original KNR JEM [5]. Revisions included backcasting over the 1910–73 time period and the development of nickel speciation fractions and levels by department and time period ([5].pp340). We examined the effect of the revisions on the cumulative exposures to nickel species [mg m^-3 ^yr] predicted by the ICNCM and Grimsrud *et al*. JEMs for a hypothetical KNR worker employed continuously over successive 10 year postwar periods in key categories of work/departments (Table 7) (Table 8). We performed this analysis knowing that correlation and regression analyses examining dose-response relationships between nickel exposure and lung cancer risk would apportion risk for a worker whose job experience fell within a specific category of work and time period according to the *absolute *and *relative *values of exposure to each nickel species predicted by the JEM for that time and place. Statistically speaking, the revised absolute and relative exposures would affect estimates of lung cancer carcinogenic potency for the risk in each JEM cell defined by department and time period.

**Table 7:** Total exposure to nickel and its species [mg Ni/m^3 ^yr] predicted by ICNCM (1990) [3] and Grimsrud *et al*. (2000) [5] JEMs for a hypothetical KNR worker with 10 years of continuous postwar employment by time period & job category

		**Nickel exposure by species and total [mg Ni/m^3 ^yr]**									
		
		**ICNCM (1990)^a^**					**Grimsrud et al. (2000)^b^**				
**Category of work**	**Time period^c^**	**Metallic**	**Oxidic**	**Sulphidic**	**Soluble**	**Total**	**Metallic**	**Oxidic**	**Sulphidic**	**Soluble**	**Total**
Roasting (day workers)	1946–1955	3.0	100.0	3.0	0.0	106.0	1.2	29.0	6.0	4.0	40.3
	1956–1965	3.0	50.0	3.0	0.0	56.0	0.9	20.5	4.3	2.9	28.5
	1966–1975	3.0	50.0	3.0	0.0	56.0	0.8	18.6	3.9	2.6	25.8
	1976–1985	0.6	12.4	3.0	0.0	16.0	0.1	5.7	0.8	0.9	7.5

Old smelter bldg. no. 1 (day workers)^c^	1946–1955	13.0	100.0	3.0	0.0	116.0	5.7	26.1	1.6	3.7	37.0
	1956–1965	13.0	50.0	3.0	0.0	66.0	4.3	16.1	0.9	2.4	23.7
	1966–1975	5.0	12.4	11.0	0.0	28.4	3.7	14.0	0.8	2.1	20.6

Calcining, smelting	1946–1955	0.0	50.0	3.0	0.0	53.0	0.4	31.1	1.9	3.7	37.0
	1956–1965	0.0	50.0	3.0	0.0	53.0	0.2	20.6	1.2	2.4	24.5
	1966–1975	0.0	50.0	3.0	0.0	53.0	0.2	17.7	1.1	2.1	21.1
	1976–1985	0.0	12.4	3.0	0.0	15.4	0.1	6.2	0.8	0.9	8.0

Nickel electrolysis^d^	1946–1955	0.0	3.0	3.0	13.0	19.0	0.0	0.1	0.1	1.5	1.7
	1956–1965	0.0	3.0	3.0	13.0	19.0	0.0	0.1	0.1	1.5	1.7
	1966–1975	0.0	3.0	3.0	13.0	19.0	0.0	0.1	0.1	1.4	1.6
	1976–1985	0.0	0.6	0.6	5.0	6.2	0.0	0.1	0.0	0.9	1.1

Copper leaching	1946–1955	0.0	13.0	0.0	13.0	26.0	0.2	7.4	0.2	7.4	15.0
	1956–1965	0.0	13.0	0.0	13.0	26.0	0.1	4.9	0.1	4.9	10.1
	1966–1975	NA	NA	NA	NA	NA	0.1	4.4	0.1	4.4	9.0
	1976–1985	NA	NA	NA	NA	NA	0.0	1.7	0.0	1.7	3.4

Copper cementation^e^	1946–1955	13.0	13.0	0.0	13.0	39.0	5.3	0.6	0.6	5.3	11.8
	1956–1965	13.0	13.0	0.0	13.0	39.0	5.2	0.6	0.6	5.2	11.5
	1966–1975	13.0	13.0	0.0	13.0	39.0	4.7	0.5	0.5	4.7	10.6

^a ^Time periods and exposure levels by nickel species are given in Table six in ICNCM (1990) [3], which separates exposure levels during 1946–1967 for Roasting day workers, i.e. Roasters (Group 2b) and Smelter building number 1 (Gp.2c), into a very high exposure period (1946–1955) and a high period (1956–1967). JEM values for 1976–84 in ICNCM (1990) [3] were extended to 1985 in this table. ^b ^Exposure levels for total nickel and nickel fractions over time periods are taken from Table three and Figure one in Grimsrud *et al*. (2000) [5]. ^c ^Applicable time periods for nickel fractions in old smelter building No. 1 are shown in Table three of Grimsrud *et al*. (2000) [5] as 1930–1950 and 1951–1977. ^d ^References to the nickel electrolysis dept. in Grimsrud *et al*. (2000) [5] and to the nickel tankhouse dept. (Group 4e) in ICNCM (1990) [3] are assumed equivalent. ^e ^Applicable time period for nickel fractions in copper cementation is shown in Table three of Grimsrud *et al*. (2000) [5] as 1927–1977. NA: Not Applicable (i.e. JEM values for the entire period were either not published or not applicable).

**Table 8:** Relative exposure to nickel species [%] predicted by ICNCM (1990) [3] and Grimsrud *et al*. (2000) [5] JEMs for a hypothetical KNR worker with 10 years of continuous postwar employment by time period & job category ^a^

		**Nickel exposure fractions by species [%]**							
		
		**ICNCM (1990)**				**Grimsrud et al. (2000)**			
**Category of work**	**Time period**	**Metallic**	**Oxidic**	**Sulphidic**	**Soluble**	**Metallic**	**Oxidic**	**Sulphidic**	**Soluble**
Roasting (day workers)	1946–1955	3	94	3	0	3	72	15	10
	1956–1965	5	89	5	0	3	72	15	10
	1966–1975	5	89	5	0	3	72	15	10
	1976–1985	4	78	19	0	2	76	10	12

Old smelter bldg. no. 1 (day workers)	1946–1955	11	86	3	0	15	70	4	10
	1956–1965	20	76	5	0	18	68	4	10
	1966–1975	18	44	39	0	18	68	4	10

Calcining, smelting	1946–1955	0	94	6	0	1	84	5	10
	1956–1965	0	94	6	0	1	84	5	10
	1966–1975	0	94	6	0	1	84	5	10
	1976–1985	0	81	19	0	1	78	10	11

Nickel electrolysis	1946–1955	0	16	16	68	1	8	5	86
	1956–1965	0	16	16	68	1	8	5	86
	1966–1975	0	16	16	68	1	8	5	86
	1976–1985	0	10	10	81	2	10	4	84

Copper leaching	1946–1955	0	50	0	50	1	49	1	49
	1956–1965	0	50	0	50	1	49	1	49
	1966–1975	NA	NA	NA	NA	1	49	1	49
	1976–1985	NA	NA	NA	NA	1	49	1	49

Copper cementation	1946–1955	33	33	0	33	45	5	5	45
	1956–1965	33	33	0	33	45	5	5	45
	1966–1975	33	33	0	33	45	5	5	45

^a ^Percentages are calculated for each group of nickel exposures shown in Table 7, identified by species, category of work, time period and ICNCM (1990) [3] or Grimsrud *et al*. (2000) [5] study. Data may not sum to 100 due to rounding error. NA: Not Applicable.

The JEM changes by Grimsrud *et al*. [5] (shown in Table 7) produced enormous reductions in nickel exposure across all species, categories of work and time periods (e.g. 80–90% reduction in total exposure in the nickel electrolysis category). On the other hand, relative exposure to soluble nickel was increased in 4 of 5 categories of work (copper leaching excepted) by reducing relative exposure to oxidic nickel in those categories. In four departments [roasting (day workers), old smelter building no. 1 (day workers), copper leaching and copper cementation], sulphidic nickel levels increased, dropping only in nickel electrolysis (shown in Table 8).

The reductions in KNR's historical exposure values had the effect of increasing lung cancer risk (per unit dose) for all nickel species in dose-response modeling studies. The effect of increasing relative soluble nickel exposures and decreasing relative oxidic nickel exposures was to increase soluble nickel's share of the overall risk at the expense of oxidic nickel's share. The absence of a systematic and protocol-driven procedure for these revisions meant that, unlike the original KNR JEM, it was impossible to test the validity and reliability of the resulting exposure dataset's effect on risk estimates in subsequent modeling studies. In the ICNCM JEM, averaging created a systematic upward bias in absolute exposure values, whose effect on risk estimation could have been studied. In our opinion, this is not possible with the latest KNR JEM and obscures the search for the sources of lung cancer risk in the refinery.

Without access to the complete KNR epidemiological database, it is impossible to reach precise conclusions. However, this preliminary examination strongly suggests that the overall effect of KNR JEM changes by Grimsrud *et al*. [5] was to *increase *soluble nickel's share of the overall risk of lung cancer in the refinery. This increase came in key departments [i.e. roasting and smelting, and electrolysis] identified in a succession of KNR studies from Pedersen *et al*. (1973) to Grimsrud *et al*. (2000) [1-5] as the principal sources of the refinery's lung cancer risk. Furthermore, it appears that the increase in risk attributed to soluble nickel exposures came primarily at the expense of oxidic nickel since this latter species' hypothesized share of carcinogenic risk declined. The rationale provided by Grimsrud *et al*. (2000) [5] to justify changes to the original ICNCM job exposure matrix and its use of backcasting procedures to fill in the empty portions of the refinery's exposure history back to the 1910 start date lack a sound scientific basis. Part of this rationale hinges on a key environmental study by Andersen et al. (1998) [26] that is shown in the next section to be scientifically unsound. This finding calls into question the validity of inferences drawn in Grimsrud *et al*. (2002, 2003, 2005) that were based on the revised JEM [6-8].

Table 9 from the Andersen *et al*. (1996) [4] and Grimsrud *et al*. (2003) [7] follow up studies displays KNR lung cancer risk by year of first exposure and time since first exposure. The studies share several features. For workers with 15+ years since first exposure, risk in every subgroup defined by year of first exposure was significantly elevated and declined in time *except *in the most recently hired subgroup (1968–83) where it reversed direction, reaching nearly the same level as in pre-WWII workers in Andersen *et al*. (1916–44) and exceeding the earliest group's risk in Grimsrud *et al*. [7]. These findings are *counterintuitive *since KNR environmental exposures have been steadily declining in time [F4] [F6], and points once again to misclassification issues in the epidemiological data. In both studies, the reader can note the mostly non-significantly elevated risk in workers with 1–14 years since first exposure (and upturn in risk for the most recent subcohort yet again), suggesting that these men were entering the workforce with prior lung cancer risk.

**Table 9:** Risk of lung cancer among KNR workers by year of first exposure and time since first exposure in Andersen *et al*. (1996) [4] and Grumsrud *et al*. (2003) [7] studies^a^

**Year of first exposure**	**Time since first exposure (yr)**								
	
	**1–14**			**15+**			**Total**		
	
	**Obs**	**SIR**	**95% CI^b^**	**Obs**	**SIR**	**95% CI**	**Obs**	**SIR**	**95% CI**
Andersen et al. (1996):									
1916–44	0	-		30	440	300–630	30	470	320–670
1945–55	7	220	90–450	95	330	270–400	102	320	270–390
1956–67	5	180	60–420	28	280	190–400	33	260	180–360
1968–83	6	230	80–490	11	410	200–730	17	320	180–510

Grimsrud et al. (2003):									
1910–29	NA^c^	-	-	17	480	280, 770	17	480	280, 760
1930–55	10	250	120, 460	160	270	230, 310	170	270	230, 310
1956–78	8	110	50, 220	67	250	190, 310	75	220	170, 270
1979–89	2	240	30, 880	3	580	120, 1690	5	370	120, 870

^a ^From Table three in Andersen et al. (1996) [4] and Table two in Grimsrud *et al*. (2003) [7]. ^b ^CI, confidence interval. ^c ^NA, not applicable.

In a recent e-letter, Andrews and Heller (2006) published an analysis of the Grimsrud *et al*. (2002) [6] case control study [44], which used the revised JEM, to demonstrate that smoking and nickel exposure were strongly related in their study, making it impossible to assess the risk from exposure. Appendix 2 lists the SAS^® ^program code for our analysis (see Appendix 3 for additional explanatory material). The principal author replied by dismissing our concerns [45]. However, the counterintuitive relationship between risk and year of first exposure and the entrenched prior risk in new hires discussed above reinforce the conclusions in our analyses showing smoking and nickel exposure interaction in the most recent study.

### 3. KNR environmental studies

Concern about the levels of soluble nickel exposure in KNR's electrolysis and RSC departments was noted in the Preface to the ICNCM report ([3].pp5–6); and led to a 1998 speciation study at the refinery [26]. Its purposes were: to investigate if workers in the RSC department were exposed to soluble nickel, to demonstrate a speedier method for speciation than the Zatka *et al*. (1992) industry standard [46], and to confirm the presence of soluble nickel compounds by other analytical methods. This study was problematic by its very nature. For example, it assumed the same type of roasting was taking place in KNR's new fluid bed furnaces as in its old multi-hearth Herreshoff furnaces (replaced by 1978). Process feeds and kinetics of roasting for the two furnace technologies are, however, very different. The newer roasting uses a copper sulphide residue after leaching most of the nickel with chlorine [47], which is not at all like the multi-hearth roasting where the feed was a nickel-copper sulphide matte. Not only are the feeds different for the two furnace types; the roasters themselves are very different. The old multi-hearth had a well controlled temperature gradient to prevent caking and sintering as the feed fell in stages from top to bottom. In contrast, the fluid bed is indeed fluidized and, therefore, much more homogeneous in temperature. Therefore, the kinetics and chemistry of the roasting processes in the two furnace types is expected to be significantly different. Furthermore, the amounts of dust leaking out of the older multi-hearth roaster far exceeded dust leakages from a fluid bed roaster. For these reasons, therefore, it made no scientific sense to design a study to collect samples from the four floors and basement of the new roaster building when the old Herreshoff furnaces no longer existed. One could reasonably hypothesize that each floor accessing a different height of a multi-hearth roaster would have differences in dust reflecting differences in the chemistry and temperatures at each level of the roaster, and this fact would be reflected in aerosol sample differences. However, these conditions would not apply in a modern fluidized bed roaster. The authors gathered data to measure roaster conditions that no longer existed!

The sampling methods were also of concern. Five parallel sets of stationary samples were collected for each floor and the basement for a total of 25 samples using an airflow rate of 20 m^3 ^d^-1 ^over 3–6 days. This procedure yielded dust samples from each filter weighing 50–100 mg. These sampling methods can be compared with those in the Werner *et al*. (1999) studies, also conducted at the same refinery and time period, that measured inhalable and total aerosol exposures for four different process areas including roasting/smelting processes [48,49]. The latter studies used personal aerosol samplers mounted on a lapel in the worker's breathing zone for a full work shift, where possible, but for four hours at least at flow rates of 2 L min^-1^. The sample measurements gathered from the roasting/smelting process (using 37 mm cassette samplers) averaged 0.12 and 0.10 mg m^-3 ^of inhalable and 'total' aerosol exposures, respectively. At the sampling rates used by Andersen *et al*., Werner *et al*. would have had to operate their samplers for 21–42 days to filter the same volume of air as the former team (1 L min^-1 ^= 1.44 m^3 ^d^-1^) and would have collected 3.6–7.2 mg of inhalable and 3–6 mg of total aerosol exposures, respectively. The differences in sampling methods in the two studies are also, therefore, of concern.

We asked Dr. Vladimir Zatka, a former research chemist with Inco Ltd., to comment on Andersen *et al*. (1998) [26] [F9: Zatka VJ: Comments on: Andersen I, Berge SR, and Resmann F: Speciation of airborne dust from a nickel refinery roasting operation. Analyst 1998; 123: 687–689. 2005. Available from Vale Inco Ltd.]. He noted that it would be impossible for the authors to guarantee sampling homogeneity, i.e. to know whether the chemical composition of the dust collected on day 1 was the same as on day 6. For his speciation method, Zatka's dust samples averaged about 2 mg in order to ensure that the speciated nickel phases never fell below the limits of detection of atomic absorption spectrometry (2 μg per filter). As an analytical chemist, his rule of thumb was to never work with samples greater than 10 mg. Even if the solid phase on a filter in the Andersen *et al*. study were at room temperature, he and Conard *et al*. (2008) [50] noted that oxygen and water in the air swept through the particles on a filter could cause oxidation and sulphate formation, changing the values estimated for the nickel phases.

The Andersen *et al*. [26] study samples were separated into two groups so that an external laboratory could apply the speciation method developed by Zatka *et al*. (1992) [46] as a check on the modified method that was proposed by the authors to provide rapid measurements of two phases only, soluble and insoluble nickel. The speciation results for all floors but one overestimated the soluble nickel percentage, which Zatka attributed to the modified method's reliance on the Blauband ("Blue band") filter, which would have passed some of the finest solid particles through its relatively larger pore size.

The most startling result reported in Andersen *et al*. (1998) was the Ni:Cu ratio ([26].pp688). In the feed to the copper-sulphide roasting, the authors reported a Ni:Cu ratio of 0.17. They also reported that workroom air sample ratios ranged from 0.29 to 0.62. The authors provided no explanation for this finding, simply noting that it was an '*interesting result' *([26].pp688). Nickel dust preferentially exiting the roaster provides one highly improbable explanation. Another more likely explanation is that fugitive nickel-containing aerosols were infiltrating the workroom areas from elsewhere in the plant. This idea is difficult to dismiss because of KNR's unique contiguous and stacked plant layout features and the opportunities it provided for the migration between departments of dust generated by other refinery processes. In fact, the authors raised this idea in their introduction without pursuing it ([26].pp687):

*That report *[Pedersen *et al*. (1973)] [1]*clearly demonstrated that the risk of lung cancer was equal or even higher for workers in the Electrolysis department compared with workers in Roasting and Smelting. This was surprising and in contradiction with earlier reports and with the then prevailing view that the lung cancer risk was related to nickel dust and insoluble nickel compounds, and not to water soluble nickel sulfate and chloride. Some explained the results from the Norwegian refinery as due to 'mixed exposures', i.e., that, owing to the operational conditions there were a lot of insoluble nickel compounds also in the Electrolysis department*.

Other concurrent, well conducted studies of the atmosphere in KNR's RSC department examined the relationship between total and inhalable metal and metal compound aerosol exposures and found that the nickel species' fractions were 63% oxidic, 26% soluble and 10% sulphidic [F10: Aitken RJ and Hughson GW: Field evaluation of a multistage personal sampler for inhalable, thoracic, and respirable dust in the nickel industry. Institute of Occupational Medicine, Research Park North, Riccarton, Edinburgh, EH14 4AP, Scotland, 2004. Available from the Nickel Producers Environmental Research Association, Durham, NC]; and 81.0% oxidic, 10.3% soluble and 8.4% sulphidic ([48].pp559). Both studies reported the presence of significant fractions of known carcinogens, oxidic and sulphidic nickel, in the RSC atmosphere.

### 4. Other nickel operations with soluble nickel exposures

Estimates of lung and nasal cancer risk from the most recent studies of other nickel operations with environmental exposures to soluble nickel are depicted in the next table (Table 10). Except where noted, risk estimates did not account for the prior risk of lung cancer (from smoking or off site risky work exposures to asbestos, pesticides, etc.) by removing the first 15–20 PYRs since first exposure.

**Table 10:** Lung and nasal cancer risk in other nickel operations with environmental exposures to soluble nickel

**Location of nickel operation & Category of work**	**Variable with levels**	**Follow up period**	**Lung cancer**		**Nasal cancer**	
			
			**Obs**	**SIR/SMR**	**Obs**	**SIR/SMR**
**Clydach Wales refinery:**	Year first employed:					
All workers^a^	Before 1920	1931–1985	83	617^j, n^	55	37647^j, n^
"	1920–1929	1931–1985	88	314^j, n^	12	7255^j, n^
"	1930–1939	1931–1985	20	138^j^	1	1434^j, n^
"	1940–1949	1940–1985	14	118^j^	0	-
"	1950–1992	1950–1985	9	84^j^	0	-
All workers^b^	1953–1992	1958–2000	28	139^j, k^	1	995^j, n^
"	1953–1962	1958–2000	18	137^j^		
"	1963–1972	1963–2000	10	156^j^		
"	1973–1992	1973–2000	0	0		
**Port Colborne Ontario refinery:^c, d^**	Duration of exposure:					
LC&S workers	≥ 5 years	1950–1984	38	366^j, n^	15	17045^j, n^
"	25+ years	"	7	363^j, n^	0	-
"	Total	"	72	241^j, n^	19	7755^j, n^
Non-LC&S workers	≥ 5 years	"	29	97^j^	0	-
"	25+ years	"	17	89^j^	0	-
"	Total	"	30	93^j^	0	-
Nickel anode work^p^	"	"	7	91^j^		
Electrolytic work^p^	"	"	23	99^j^		
Yard/Transportation work^p^	"	"	21	87^j^		
**Harjavalta Finland smelter & refinery:^e^**						
All nickel exposed workers	Latency 20+ years	1953–1995	20	212^i, m^	2	1590^i, n^
Smelter workers	Latency 20+ years	"	13	200^i, l^	0	-
"	5+ years exposed	"	8	101^i^	0	-
"	<5 years exposed	"	7	250^i, l^	0	-
Refinery workers	Latency 20+ years	"	6	338^i, m^	2	6710^i, n^
"	5+ years exposed	"	3	199^i^	2	7520^i, n^
"	<5 years exposed	"	3	375^i, l^	0	-
**Thompson Manitoba refinery:^f, g^**	Year first employed:					
All workers	1960–1986	1960–1986	25	116^j^	0	-
Salaried workers	1960–1986	1960–1986	5	155^i^	0	-
Miners (hourly workers)	1960–1986	1960–1986	7	96^j^	0	-
Smelter workers (hourly)	1960–1986	1960–1986	4	155^i^	0	-
Refinery workers (hourly)	1960–1986	1960–1986	6	172^j^	0	-
**British nickel plating company:^h^**	Year first employed:					
All workers	1945–1975	1945–1993	11	108^j^	0	-

^a ^Easton *et al*. (1992) [13]. ^b ^Sorahan *et al*. (2005) [21]. ^c ^Roberts *et al*. (1989) [9,10]. ^d ^15+ years since first exposure. ^e ^Antilla *et al*. (1998) [23]. ^f ^Roberts *et al*. (1991) [F1]. ^g ^Male workers with 15+ years since first exposure. Incidence ratios include salaried & hourly workers; mortality ratios include hourly workers. ^h ^Pang *et al*. (1996) [24]. ^i ^SIR- Standardized Incidence Ratio. ^j ^SMR- Standardized Mortality Ratio. ^k ^p < 0.1. ^l ^p < 0.05. ^m ^p < 0.01. ^n ^p < 0.001. ^p ^Included all men who ever worked in the given category from their first date of work in that category. CI-Confidence Interval. LC&S-Leaching, Calcining and Sintering work. Non-LC&S work included the electrolysis and nickel anode departments, yard and transportation work.

For Clydach workers, lung cancer risks were significantly elevated among men first employed during the operation of the copper plant and hydrometallurgical departments (before 1937), a period coinciding with arsenic contamination in the environment (see next section). However, by the 1930's, risk for this inception cohort had fallen to levels consistent with higher putative smoking prevalence in the workforce (defined by the ICNCM's chair as an SMR with a lower bound on the 95% CI under 150) ([3].pp6). Clydach epidemiologists have noted that '*the greatest change in exposure to a known carcinogen that occurred over this period *[<1910–1924] *was, of course, the increase in cigarette smoking, and national lung cancer rates in Britain increased by an order of magnitude over the period spanned by the different birth cohorts in *[the Peto *et al*. (1984) refinery] *study' *([19].pp44). No published human health study of this workplace, to our knowledge, has ever taken smoking risk in its workforce into account [F11: Warner JS: Comments on IDSP Report No. 12: Lung cancer in the hardrock mining industry. Submission to the Ontario Workers' Compensation Board (WCB), September 1994. Also, Warner JS: Addendum Submission to WCB, July 1995. Available from Vale Inco Ltd.]. One nasal cancer was reported in the 1930s decennial cohort in Easton *et al*. (1992) [13] and the 1953–92 cohort in Sorahan *et al*. (2005) [21], the latter of which was not a nasal primary tumour. [Note to the reader: Smoking prevalence would not be expected to differ substantially among the same process workers in the company's Clydach and PCNR facilities. See related letter on this subject in Sorahan T and Williams SP: Respiratory cancer in nickel carbonyl workers [Letter]. *Occup Environ Med *2006; 63: 856.]

For PCNR workers, significantly elevated lung cancer risk was found only in the Leaching, Calcining and Sintering (LC&S) category of work where high levels of sulphidic and oxidic nickel were present. LC&S workers acquired risk with as little as 1 year of exposure (SMR = 183) [10]. None was found in other (Non-LC&S) departments, including electrolysis work with its predominantly soluble nickel exposures. The ICNCM report examined the PCNR cohort data and found no evidence of lung cancer risk that could be attributed to electrolysis work, but did find nasal cancer risk among electrolysis workers with less than 5 years of LC&S work. However, no nasal cancers occurred in a subgroup with 15 or more years since first electrolysis department exposure who had "high" soluble nickel exposure (from the washing of anode scrap and pumping of anode slimes) and less than 5 years of LC&S work ([3].pp56). This same subgroup's lung cancer risk, however, was elevated with 6 observed and 2.51 expected deaths, 12% and 7%, respectively, of the observed and expected lung cancer deaths among all PCNR workers with less than 5 years in LC&S work. The report noted that only 5.6% (N = 109) of the electrolysis workers had any exposure in these areas, and only 1.3% (N = 25) had more than five years exposure. The ICNCM's authors acknowledged that sintering work in this subgroup weakened their argument for soluble nickel risk.

The latest Harjavalta study [23] found elevated lung cancer risk in nickel smelter and refinery workers with 20+ years since first exposure (SIR = 200 and 338, respectively). However, the risk in smelter operations (not considering latency) was confined to workers with less than 5 years exposure (SIR = 250), and none was found in the 5+ years of exposure group (SIR = 101). Although lung cancer risk in the refinery workers (not considering latency) was elevated in both the <5 years and 5+ years exposure groups (SIR = 375 and 199, respectively), the highest risk was again found in the group with least duration of exposure. This declining gradient of lung cancer risk (with increasing years of exposure) in both smelter and refinery workers suggests employee misclassification, possibly related to: the assignment of men who worked at two or three refinery sites in all categories ([23].pp246), sulphuric acid mist exposure (see following), and smoking-related confounding. Nasal (and stomach) cancer risk was found in refinery workers with 20+ years since first exposure (2 and 3 cases, respectively) and with 5+ years of exposure (2 and 4 cases, respectively).

No account was taken of sulphuric acid mist exposure, a Group I carcinogen [51], in the leaching of nickel matte and electrowinning processing at Harjavalta [52]. The wearing of protective breathing apparatus in the refinery's electrowinning halls became mandatory *only *in 1990 and was not widely observed until 1993. Recent H_2_SO_4 _stationary measurements in the halls ranged in average as follows [mg m^-3^]: 0.64–1.05 (2003); 0.04–0.56 (2004); 0.18–0.56 (2005); and 0.06–0.67 (2006). The current Occupational Exposure Limit (OEL) for this substance is 0.2 mg m^-3 ^but was previously set at 1.0 mg m^-3 ^[F12: Rantanen T: Personal communication, Nov. 28, 2006. Available from Outokumpu refinery, Harjavalta Finland].

The Thompson refinery study [F1] found slightly elevated lung cancer risk in smelter and refinery workers, but the small number of cases, young cohort (mean age at hire: 24 years) of short stay workers (mean service: 4.3 years) and short follow up (mean follow up: 17.4 years) necessitate continued follow up of the workforce.

For the sake of completeness, a mortality study of British nickel platers was reviewed [24]. Workers in the chromium plating or nickel/chromium plating departments were excluded, leaving 284 men who received nickel chloride and nickel sulphate aerosol exposures in the nickel plating departments. Stomach cancer was the only reported diagnosis with elevated risk (8 observed and 2.49 expected deaths).

### 5. Arsenic as a source of carcinogenic risk in nickel production

The role of arsenic, a Group I carcinogen [53], as an agent of historic occupational cancer risk in the nickel industry has never been adequately investigated despite case reports as early as 1939 of arsenic induced illness [14]. Arsenic is often found in nickel ore bodies, and where it appears as orcelite, a complex defect structure of Ni_5-x_As_2_, nickel arsenide, it is best represented chemically as (Ni, Fe, Cu)_4,4-4.2_(As, S)_2 _to indicate that Fe and Cu can and do substitute for nickel and sulphur substitutes for arsenic [F13: Conard BR: Personal communication, August 7, 2003. Available from Vale Inco Ltd.]. Arsenic has accompanied nickel exposures historically in various steps of nickel production.

From 1901 to 1934, pre-reduction nickel oxide at the Clydach refinery was produced by calcining a feed stock known as Bessemer matte that was imported from Canada. [Note to the reader: From 1903 to 1930, the Clydach refinery received sulphidic Bessemer matte imported from the Coniston smelter in Sudbury, Canada from which copper was leached with sulphuric acid. This process was phased out from 1930 to 1936.] This was a high nickel, high copper sulphide mixture (45% Ni, 35% Cu, 16% S). After calcining, a large fraction of the copper was leached out with dilute sulphuric acid (~10%) and, after recrystallization, marketed as copper sulphate. This was a large operation involving tens of thousands of tonnes of sulphuric acid per annum. The copper-depleted calcine was transferred to a closed system of sequences of towers for the reduction, carbonylation and decomposition steps for the removal of nickel. The residue comprised some 20% of the original charge, but was still relatively rich in nickel, cobalt and precious metals. This concentrate then underwent sulphidization using gypsum, sand and coke in a batch furnace operation. Several batches were combined, calcined, leached again to remove copper oxide, reduced and carbonylated to enable further nickel recovery. This process was repeated up to seven times to recover more nickel and commercially significant quantities of cobalt and precious metals. What was not appreciated until 1920 was the fact that use of a cheap source of sulphuric acid contaminated with significant quantities of arsenic was a contributor to the inefficiency of nickel extraction. Furthermore, this unwanted arsenic was successively concentrated in the recycling processes, reaching 8–10% in later batches as determined recently in analyses of two samples of Clydach process materials in powder form dating back to 1920 and 1929. Analysis of 12 elements in the samples revealed significant differences only for arsenic and iron. The 1920 sample contained 9.6% of arsenic and 4.4% of iron while the 1929 sample had 1.0% and 0.8% respectively. Both samples contained arsenic in the form of the compound orcelite. It appears to have been formed by interactions occurring, most probably, in the furnacing operations. Draper (1997) remarks that the presence of arsenic in the process materials was well-known and some concern about the medical implications was expressed by the medical staff, because there was some evidence of arsenicism among process workers [14,27]. Also, the sample particles were of respirable size, averaging 2 μm in diameter. The presence of arsenic contamination in Clydach's refining processes during the 1902–1934 period has been hypothesized to account for much, if not all, of the observed respiratory cancer risk during this time. From 1932 to 1936, the entire calcine-leaching-copper sulfate production-concentrate recycling was eliminated and the Bessemer matte feedstock was replaced with low copper, low sulphur feedstocks [27].

To address the Clydach refinery arsenic hypothesis, Draper (1997) reconstructed detailed work histories for the 365 respiratory cancer cases (280 lung and 85 nasal cancers) attributed to exposure during the 1901–1970 period. He found that 81 of the 85 nasal cancer cases and 260 of the 280 lung cancer cases began work during the high risk period before 1928. The work records of 215 lung and 85 nasal cancer cases showed that their critical exposures all occurred either from processes operating until the mid-1930's or from contaminant residues lingering in machinery or buildings from the operations of the first four decades. The seven high risk job designations in these work histories included 4 major sequential process sites in the winning of nickel metal (calciner, copper shed, nickel shed and furnaces), 2 main subsidiary process sites dependent on the main process line (copper sulphate and nickel sulphate), and rigger/fitter/handyman (the skilled technicians and tradesmen that maintained or rebuilt the machinery of the production lines) [27].

In the all-sulphate system in use at KNR up until about 1953, it was necessary to treat the anolyte with reduced matte to neutralize excess acid and precipitate the copper by cementation. The arsenic was also precipitated in this step and was recycled to the roasters with the cement copper. Arsenic was thus allowed to build up in the refinery circuit to as high a concentration as could be tolerated without contamination of the final products, and was bled from the system by the periodic removal of cement copper (containing 10.4% As by weight) (Table 11) ([3].pp17). Cementation was carried out in 20 mechanically agitated tanks. We noted elsewhere that '*even though the reduced matte was delivered in a moist condition, the feeding operation was reported to be one of the dirtiest in the plant*.' KNR electrolysis workers, therefore, were likely exposed to nickel arsenide dust made airborne by passage of heated air through a bed of cement copper slimes located in the Electrolysis Department for drying in preparation for return (by ER personnel) to the roasters. After conversion to a predominantly chloride circuit in 1953, it was advantageous to precipitate iron before cementation, resulting in a reduction in arsenic (in the cement copper step) from 10.4% As by weight before 1953 to 0.3% afterwards (although As was eliminated from the circuit after 1953 primarily in the Fe precipitate step containing 4.0% As) ([F2].pp14).

**Table 11:** Typical analyses of KNR solids and electrolytes*

	**Weight^a^**						
**Product**	**Nickel**	**Cobalt**	**Copper**	**Iron**	**Arsenic before 1953**	**Arsenic after 1953**	**Sulphur**
Matte as received	48	1.0	28	1.5	0.2	0.2	22
Cement copper slime	32	-	35	3.0	7.5	0.6	-
Cement copper	13	-	68	1.7	10.4	0.3	-
Herreshoff calcine	44	1.0	32	1.7	2.4^d^	0.2	0.7
Leached matte^b^	58	1.2	15	1.9	3.4^d^	0.3	0.9
Reduced matte^b^	71	1.3	19	1.5	4.0^d^	0.3	1.7
Nickel anodes	75	1.5	17	1.6	3.7^d^	0.3	1.1
Raw anode slime	30	0.8	27	4.5	3.0^d^	0.1	21
Roasted anode slime	36	0.9	30	5.0	2.0	0.1	1.1
Iron precipitate	1.2	-	1.2	39	0.4	4.0	-
Copper electrolyte	70	4.0	75	-	-	-	-
Nickel anolyte^c^	68	0.2	2.3	0.4	0.4	0.03	-
Nickel catholyte^c^	68	0.2	Tr^e^	Tr^e^	Tr^e^	Tr^e^	-

* Revised from Table eight in ICNCM (1990) [3]. ^a ^Composition of copper electrolyte, nickel anolyte, and nickel catholyte in grams per liter. Composition of other products expressed as percentage by weight. ^b ^So named for convenience. Actually "leached matte" is "leached calcine" and "reduced matte" is "reduced leached calcine." ^c ^Nickel electrolyte contained 160 g of nickel sulphate per liter without any nickel chloride before 1953. After 1953, most of the nickel sulphate was replaced by 95 g of nickel chloride per liter, leaving only 45 g of nickel sulphate per liter. ^d ^Revised by P. Thornhill. ^e ^Tr = trace.

The issue of lung cancer risk and arsenic exposures at KNR was recently addressed in Grimsrud *et al*. (2005) but relied on the revised JEM described above for its analysis, one of the several reasons that undermine the study's findings associating excess risk with water soluble nickel exposure [8].

Although arsenic is present in the mined nickel ores in the Sudbury basin, no systematic measurements were ever reported. Nevertheless, some published data exist. The basin's Frood and Garson mines provided arsenic-rich nickel ores for the Coniston sinter plant where significantly elevated lung cancer risk (SMR = 298) was recorded [10]. Until 1934, Coniston's bessemer matte in which the concentration of arsenic as an arsenide was about 0.2%, was Clydach's feedstock [27]. Falconbridge's arsenic-rich nickel mine in the basin (where elevated As levels in soil were recently detected in a risk assessment proceeding under regulatory authority) provided its sinter plant's feedstock where elevated lung cancer risk was reported (SMR = 144) [28]. This plant's nickel matte was shipped to KNR for final processing. In both sinter plants, sintering *preceded *the smelting step. In contrast, sintering *followed *the smelting step in both the Copper Cliff (CC) sinter plant and PCNR's LC&S department where lung and nasal cancer risks were significantly elevated (p < 10^-7^) [10,29]. Before 1956, all nickel sulphide destined for electrolytic refining at CC was sintered to oxide, reduction smelted, and cast into metal anodes. To avoid preferential fusion rather than oxidation, the sulphide feed was diluted with five times its weight of sinter returns [54], undoubtedly magnifying dust levels for all contaminants, including arsenic, enormously and causing the significant respiratory carcinogenic risk reported by Chovil *et al*. (1981), among others, for this plant before the process was changed in 1962 [29]. This study found 57 cases of lung cancer and 7 of nasal cancer in a cohort of 495 workers, all of whom were employed at CC between 1948 and 1962. All 64 cases were first employed before 1957; and the reported SIRs (standardized incidence ratios) were 10.71 and 1.85 for men first employed in 1948–51 and 1952–62 respectively. We contend that enough published evidence has existed for some time to establish the hypothesis of arsenic's contribution to the overall historical respiratory cancer risk recorded at CC, Coniston, the Falconbridge sinter plant, PCNR's LC&S department, Clydach and key KNR departments including electrolysis; and warrants further investigation.

### 6. Review of the toxicology of soluble nickel

The US Environmental Protection Agency (EPA) issued a nickel health advisory document in 1986 to signal speciation as a leading regulatory concern in the determination of nickel's carcinogenic potential [33]. This concern led to the creation of the ICNCM (discussed earlier) whose report in 1990 [3] concluded that more than one form of nickel can give rise to lung and nasal cancer and that much of the respiratory cancer risk seen among nickel refinery workers could be attributed to exposure to a mixture of oxidic and sulphidic nickel at very high concentrations (≥ 10 mg Ni/m^3^). The ICNCM report also concluded that there was evidence that soluble nickel exposure increased the risks of these cancers and that it may enhance risks associated with exposure to less soluble forms of nickel. It also reported that no evidence was found that metallic nickel was associated with respiratory cancer risks. The ICNCM looked for support for its findings to animal carcinogenesis studies then underway using inhalation as the route of exposure for nickel subsulphide, high temperature ("green") nickel oxide and nickel sulphate hexahydrate. It also looked to future work on the mechanisms of nickel carcinogenesis to help unify and explain its findings and those from animal experimentation.

Although not related directly to respiratory cancer risk, we note in passing that newly published studies using a population based birth and perinatal registry for the Arctic town of Monchegorsk, Russia where a nickel refinery is located found no negative effect of maternal exposure to water-soluble nickel on the risk of delivering a newborn with malformations of the genital organs [55-57].

A clearance study by Benson *et al*. (1994) [42] demonstrated a retention half-life of 4 days for nickel subsulphide and 120 days for green (high temperature) NiO exposure in inhalation studies of F344/N rats. The Ni_3_S_2 _study also detected nickel in kidney and other extrarespiratory tract tissue indicating that its clearance was dominated by a dissolution rather than a mechanical clearance pathway. Nickel was not distributed to other extrarespiratory tract tissue in the NiO study. Benson *et al*. (1995) [43] found that approximately 99% of the inhaled nickel sulphate in rats exposed to the same levels as in the NTP studies (described below) cleared with a half-time of 2 to 3 days. In mice, 80–90% of the inhaled nickel sulphate cleared with a half-time within 5 to 17 days. Nieboer reported a half-life for water soluble Ni(II) salts in the blood stream of 24 hours or less [58].

Two year National Toxicology Program (NTP) inhalation studies of male and female F344/N rats and B6C3F_1 _mice found varying strengths in the evidence of carcinogenic activity to two insoluble nickel compounds, nickel subsulphide and nickel oxide, but none at all to nickel sulphate hexahydrate, a soluble nickel compound [30-32]. A 2 year inhalation study of carcinogenicity in Wistar rats conducted by WIL Research Laboratories, Inc. showed that exposure to a lifetime dose of respirable sized metallic nickel powder did not cause cancer (Table 12) [35].

**Table 12:** Conclusions on carcinogenic activity of 2-year inhalation studies of male and female F344/N rats and B6C3F_1 _mice exposed to nickel subsulphide, nickel oxide and nickel sulphate hexahydrate [30-32]; and Wistar rats exposed to nickel metal powder [35]

		**Evidence of carcinogenic activity**			
		
		**F344/N rats**		**B6C3F_1 _mice**	
**Nickel compound**	**Ni Solubility**	**Male**	**Female**	**Male**	**Female**
Nickel subsulfide	Insoluble	Clear evidence	Clear evidence	No evidence	No evidence
Nickel oxide	Insoluble	Some evidence	Some evidence	No evidence	Equivocal evidence
Nickel sulfate hexahydrate	Soluble	No evidence	No evidence	No evidence	No evidence
		**Wistar rats**			
Nickel metal powder	Insoluble	No evidence	No evidence	-	-

The findings in these animal studies raise important questions that are addressed in Hayes' classic textbook on toxicology [37]. Dose selection plays a key issue in the design and interpretation of the animal bioassay. Typical protocols call for animal exposures at the maximum tolerated dose (MTD) and at 2–3 additional dose levels at fractions of the MTD (e.g. 1/2, 1/4, etc.). The MTD is predicted from subchronic toxicity studies as the dose *"that causes no more than a 10% weight decrement, as compared to the appropriate control groups, and does not produce mortality, clinical signs of toxicity or pathologic lesions (other than those related to a neoplastic response) that would be predicted [in the long-term bioassay] to shorten an animal's natural lifespan"*. The MTD is not a nontoxic dose and is expected to produce some level of acceptable toxicity to indicate that the animals were sufficiently challenged by the chemical. The MTD has been justified as a means of increasing the sensitivity of an animal bioassay involving limited numbers of animals so as to be able to predict risks in large numbers of humans. An objection to the use of MTDs has been that metabolic overloading may occur at high-dose levels, leading to an abnormal handling of the test compound; for example, toxic metabolites could be produced as a consequence of saturation of detoxification pathways. Organ toxicity could occur that might not happen at lower concentrations to which humans are typically exposed. Thus, it has been argued that nongenotoxic agents that are determined to be positive in rodent carcinogenicity bioassays may exert their own carcinogenicity via target-organ toxicity and subsequent cell proliferation and should not be assumed to be carcinogenic at low doses [37].

Ames and coworkers [38,39] have suggested that target-organ toxicity and subsequent mitogenesis are responsible for the fact that over half of all chemicals tested in chronic bioassays at the MTD are determined to be carcinogens in rodents. They observed that both genotoxic and nongenotoxic agents tested at the MTD cause increased rates of mitogenesis, thus increasing the rate of mutation. For several chemicals, induction of tumors was more strongly correlated with cell division than with DNA adducts or mutagenic activity. Others have reported that cancer potency and MTD are inversely correlated and that, consequently, the potency estimate is simply an artefact of the experimental design. Goodman and Wilson [40] found that cancer potency and the MTD were more strongly related for nonmutagens than for mutagens in rat bioassays, indicating that the carcinogenic effect and toxicity were more closely associated for nonmutagens than for mutagens; however, they noted that even for most mutagens, their findings suggested that at high doses carcinogenicity is induced via mechanisms associated with toxicity [37].

Gaylor [41] noted that, given sufficient animals (e.g. ~200 per group), it is estimated that about 92% of all chemicals tested would, if tested at the MTD, yield a positive response at one or more tumor sites in rats or mice. Gaylor observed that "this MTD bioassay screen is not distinguishing between true carcinogens and noncarcinogens." The author further suggests a common mechanistic explanation for this result; that is, for nongenotoxic carcinogens in particular, the mode of action involves cytotoxicity followed by regenerative hyperplasia. Thus, the relevant question is not so much whether a chemical causes cancer at the MTD (i.e., is a chemical a carcinogen?), but what is the dose at which the chemicals induce cancer [37]?

We have drawn on this toxicology literature to highlight uncertainties around the interpretation of the findings in these animal bioassays (Table 13). Cytotoxicity at the target organ (lung), i.e., chronic active inflammation and/or macrophage hyperplasia, was observed in all animals (rats and mice) exposed at all levels to all nickel groups with only a few exceptions at the lowest nickel sulphate exposure levels for both species. Yet, alveolar/bronchiolar adenomas or carcinomas were found only in rats chronically exposed to sulphidic and oxidic nickel but not to nickel sulphate and metallic nickel. And they were found only in female mice exposed to oxidic nickel (a finding rated by the NTP as 'equivocal evidence'). Cell proliferation (macrophage hyperplasia) was found in both species exposed to nickel subsulphide and nickel sulphate but not to oxidic and metallic nickel. But it was associated with lung neoplasms found only in rats exposed to nickel subsulphide and nickel oxide (for which exposure female mice also showed carcinomas) but not to nickel sulphate and metallic nickel. While the NTP exercised prudence in their conclusions, we must ask in view of the opinions noted above the following questions: (1) Were the observed cancers caused by target-organ (lung) toxicity and subsequent cell proliferation in the face of MTD levels of exposure? (2) Are these cancers likely to occur at low levels of human exposure; and (3) Were they caused by the chemical itself as carcinogen or by the dose at which the chemical(s) induce cancer?

**Table 13:** Selected neoplastic and non-neoplastic lung effects in 2 year inhalation studies of male and female F344/N rats and B6C3F_1 _mice exposed to nickel subsulphide, nickel oxide and nickel sulphate hexahydrate [30-32]; and Wistar rats exposed to nickel metal powder [35]

	**F344/N rats**		**B6C3F_1 _mice**	
**Ni species, dose and lung effects**	**Male**	**Female**	**Male**	**Female**
Nickel subsulphide:				
Dose in % of MTD^a, b^	(0, 15, 100)	(0, 15, 100)	(0, 50, 100)	(0, 50, 100)
Chronic active inflammation rate	(9/53, 53/53, 51/53)	(7/53, 51/53, 51/53)	(1/61, 52/59, 53/58)	(1/58, 46/59, 58/60)
Macrophage hyperplasia rate	(9/53, 48/53, 52/53)	(8/53, 51/53, 52/53)	(6/61, 57/59, 58/58)	(5/58, 57/59, 60/60)
Alveolar/bronchiolar adenoma or carcinoma rate	(0/53, 6/53, 11/53)	(2/53, 6/53, 9/53)^f^	None	None

Nickel oxide:				
Dose in % of MTD^c^	(0, 25, 50, 100)	(0, 25, 50, 100)	(0, 25, 50, 100)	(0, 25, 50, 100)
Chronic inflammation rate	(28/54, 53/53, 53/53, 52/52)	(18/53, 52/53, 53/53, 54/54)	(0/57, 21/67, 34/66, 55/69)	(7/64, 43/66, 53/63, 52/64)
Alveolar/bronchiolar adenoma or carcinoma rate	(1/54, 1/53, 6/53, 4/52)^f^	(1/53, 0/53, 6/53, 5/54)	None	(6/64, 15/66, 12/63, 8/64)

Nickel sulphate hexahydrate:				
Dose in % of MTD^d^	(0, 25, 50, 100)	(0, 25, 50, 100)	(0, 25, 50, 100)	(0, 25, 50, 100)
Chronic active inflammation rate	(14/54, 11/53, 42/53, 46/53)	(14/52, 13/53, 49/53, 52/54)	(1/61, 2/61, 8/62, 29/61)	(1/61, 7/60, 14/60, 40/60)
Macrophage hyperplasia rate	(7/54, 9/53, 35/53, 48/53)	(9/52, 10/53, 32/53, 45/54)	(6/61, 9/61, 35/62, 59/61)	(7/61, 24/60, 53/60, 59/60)
Neoplastic effects	None	None	None	None

	**Wistar rats**			

Nickel metal:				
Dose in % of MTD^e^	(0, 25, 100)	(0, 25, 100)		
Chronic inflammation rate	(14/50, 44/50, 41/50)	(16/50, 45/50, 45/54)		
Neoplastic effects	None^g^	None^g^		

^a ^MTD: Maximum Tolerated Dose. ^b ^MTD [Ni_3_S_2_/m^3^]: 0.73 mg (rats); 1.2 mg (mice). ^c ^MTD ["green" NiO/m^3^]: 2.0 mg (rats); 3.9 mg (mice).

^d ^MTD [NiSO_4_.6H_2_O/m^3^]: 0.11 mg (rats); 0.22 mg (mice). ^e ^MTD [Ni metal/m^3^]: 0.4 mg (rats). ^f ^Includes squamous cell carcinoma. ^g ^Oller *et al*. (2008) [35] concluded that the treatment of nickel metal powder administered by inhalation 6 h/day, 5 days/week over a two-year period did not produce an exposure-related increase in tumors anywhere in the respiratory tract, including the nose.

The NTP soluble nickel report's authors noted *in vitro *evidence [36] that water-soluble nickel (i.e. nickel chloride) enhanced the cytotoxicity and mutagenicity of DNA-damaging agents by inhibiting nucleotide excision repair in mammalian cells and repair of ultraviolet-induced photoproducts [36]. They also cited ICNCM epidemiological findings [3] involving high exposure to nickel sulphate hexahydrate (1–5 mg/m^3^) of refinery workers in the electrolysis department at Kristiansand, Norway and the hydrometallurgy department at Clydach, Wales as providing *'evidence that exposure to soluble nickel increased the risk of lung cancer in workers also exposed to oxidic, sulfidic, and/or metallic nickel'*. Both the *in vitro *and ICNCM human health studies suggested to the NTP authors that exposure to water soluble nickel may be a factor in the eventual development of cancer when there is concomitant exposure to other agents ([30].pp91). Neither the NTP nor the ICNCM report, however, labelled soluble nickel a cancer promoter.

Regulators usually require both *in vitro *and *in viv*o tests on new compounds to predict the effect in living organisms. The International Convention on Harmonization (ICH) requires a battery of 3 genotoxicity tests to be conducted on a new drug before it is given to humans in a clinical trial [59]. The ICH guidance documents now form the regulatory backbone for genotoxicity testing and assessment of pharmaceuticals in the European Union, Japan, USA and Canada. The ICH battery includes an *in vitro *test for gene mutation in bacteria; an *in vitro *test with cytogenetic evaluation of chromosomal damage with mammalian cells, or an *in vitro *mouse lymphoma tk assay; and a third test, which is actually an *in vivo *assay of chromosomal damage in rodent bone marrow cells. The inclusion of this required *in vivo *test provides a more reliable measure of genotoxicity in a whole animal; in other words, the test substance must be absorbed, metabolized and distributed to the target organ before it can produce an adverse effect. It is not possible to accurately draw inferences about genotoxicity or potential for carcinogenicity from *in vitro *short-term assays alone [F14: Goldberg MT: Response to questions arising from NTP study on nickel sulfate hexahydrate. GlobalTox International Consultants Inc., Guelph, Ontario. September 28, 2006. Available from Vale Inco Ltd.]. Variations of a 2-stage carcinogenesis test protocol pioneered by Berenblum and Shubik [60,61] form the usual basis for determining the promotional effects of a compound. That *in vivo *confirmation is lacking for soluble nickel [F14]. Nevertheless, a theory proposing its role as a carcinogenic promoter has emerged [33,34,62].

On the basis of the evidence to date, Oller *et al*. (2008) have concluded that the exact direct or indirect effects of Ni(II) ions needed for the generation of respiratory tumors are still the subject of much research. They suggest that the bioavailability of these ions at nuclear sites of target epithelial cells may determine the carcinogenic potential of Ni-containing substances. This bioavailability will depend on several factors: respiratory toxicity; deposition; clearance; target cell uptake; and intracellular dissolution (solubility) [35].

## Concluding remarks

We have adopted a weight of scientific evidence standard [63] to examine the support for the soluble nickel cancer hypothesis; and have presented new findings and new analyses of existing findings of the human health and toxicological evidence for this compound that refute or seriously weaken the proposition. Sharp contrasts in the architecture, topography, industrial hygiene, intensity of use and histories of the KNR and PCNR plants point to the likelihood of mixed insoluble nickel exposures, including arsenic, as the most probable cause of the respiratory cancer risk observed in KNR's electrolysis department; and their absence in the same environment at PCNR as the likely reason for the normal risk observed there. We have shown that misclassification problems in KNR's epidemiology are the likely cause of implausible findings of elevated respiratory cancer risk in the plant's administrative and service areas that is comparable to the observed risk in its electrolysis and RSC departments. These may be related to the existence of a part-time or seasonal workforce unacknowledged and likely, therefore, unaddressed in KNR human health studies.

We have also identified unsupported changes to historical worker exposures in the most recent KNR epidemiological studies that cast serious doubt on the validity and reliability of inferences drawn from them. Unlike the protocol driven KNR JEM developed for the ICNCM (1990) report [3], the revised JEM for all post-1998 KNR epidemiological studies lacked a systematic rationale, thereby preventing review through sensitivity analyses of the validity and reliability of the JEM changes on overall and nickel species-specific risk exposure modeling estimates. We suggest, however, that the effect of the changes would have been to *increase *lung cancer unit dose risk estimates for all nickel species, and to *transfer *risk previously attributed to oxidic nickel to soluble nickel. We also demonstrated statistically that smoking and nickel exposures were strongly related in recent KNR respiratory cancer risk studies, making it impossible to draw valid inferences on carcinogenic risk from specific nickel compounds.

The long term (2 year) NTP animal inhalation studies of soluble nickel found no evidence of carcinogenic risk. Nor has *in vivo *toxicological evidence supporting a promotional carcinogenic effect been demonstrated. In concert, the evidence from the animal bioassays for all the nickel compounds has raised several questions: (1) Were the observed cancers caused by target-organ (lung) toxicity and subsequent cell proliferation in the face of MTD levels of exposure? (2) Are these cancers likely to occur at low levels of human exposure; and (3) Were they caused by the chemical itself as carcinogen or by the dose at which the chemical(s) induce cancer?

For all these reasons, therefore, we argue that, while KNR's epidemiology has determined the overall level of historical respiratory cancer risk in the refinery, it has failed to identify accurately its causes. We suggest that close scrutiny of the Clydach epidemiological database would lead to similar conclusions. Furthermore, the era of risk attributed to soluble nickel at the Welsh refinery extended from 1902 to 1937, an era not only when rudimentary industrial hygiene practices meant that mixed exposures throughout the workplace were far more likely and when smoking behaviour was embedded in the working culture through such inducements as free cigarettes for every WWI British soldier, but also when arsenic exposures were present and likely contributed to the lung and nasal cancer risks attributed to this workplace. There is consistent historical evidence across the Clydach, KNR and all Ontario nickel processing facilities of respiratory cancer risk in the likely, but systematically unrecorded, presence of arsenic exposures. Where none of these conditions applied (viz. PCNR's electrolysis department), no evidence to support the soluble nickel-cancer risk hypothesis was found.

We know that arsenic in the ore mined in the Sudbury basin has likely found its way into all related nickel processing plants. We also know that other sources of arsenic entered processing at Clydach. We suggest that arsenic was responsible for an indeterminate proportion of the respiratory carcinogenic risk previously ascribed to one or more nickel species in refinery studies. The animal bioassay evidence was developed to address the respiratory carcinogenic potential of each of the four nickel compounds found in nickel processing environments. However, the human health evidence involved exposures to a complex mixture of nickel species and, likely, to arsenic compounds (not to ignore the contributions of other offsite risky exposures and smoking as well), and to sulphuric acid mist exposures in the Harjavalta refinery. The animal studies provide evidence for pure substance exposure conditions never found in historical refineries; and, therefore, cannot directly support propositions on nickel carcinogenicity arising from human health studies. A similar problem was identified earlier that resulted from efforts to compare the respiratory cancer risks in the KNR and PCNR electrolytic departments. Since their environmental exposures were different, their epidemiology must have differed as well. Unless the animal studies could duplicate the complex mixture of exposures found in KNR's RSC or its electrolysis department, or in the PCNR's LC&S or its electrolysis department, it could not inform the related human health evidence. This is a fundamental problem with all observational studies, and is one argument in favour of randomized clinical trials (RCTs) for epidemiological evaluations. Obviously, RCTs cannot be used to develop historical environmental and occupational health evidence, but inferences drawn from those studies must be approached with great caution.

In the absence of human health and animal evidence supporting soluble nickel's carcinogenicity, we argue that this hypothesis lacks a sound scientific basis and should be reconsidered. At the very least, an independent review should be conducted of the KNR epidemiological database to locate the source(s) of respiratory cancer risk in the refinery, whether occupational or public health or both in nature. Secondly, we argue that appropriate regulatory agencies should reconsider their recommendations concerning this nickel compound. We also note in passing that our arguments raise fresh difficulties for regulatory toxicologists dealing with the development of occupational exposure standards for all nickel compounds, particularly for the still remaining carcinogens, sulphidic and oxidic nickel.

## Appendix 1

Norwegian researchers have argued [8] [F15: Danish Environmental Protection Agency: Nickel Sulphate (CAS-No. 7786-81-4; EINECS-No. 232-104-9) RISK ASSESSMENT. Preliminary Draft, May 2002. Human Health – only. Available from Danish EPA] that the Roberts *et al*. (1989) mortality study of Sudbury and Port Colborne nickel workers [9,10] erred in finding normal lung cancer risk in PC's electrolysis workers by assuming that the 42% of the PC cohort (1,820/4,287) whose vital status had not been flagged as deceased by national record linkage methods were, therefore, still alive at the end of the study period, thereby seriously underestimating mortality rates (Table 14). Roberts *et al*. tested this assumption in two ways; first, by noting that the national record linkage methods successfully recognized death in 92% of the 5,932 study subjects known from company records to have died. The death certificates for the remaining 8% were traced manually. As a second test of record linkage, the 1989 study authors conducted an independent follow-up of 1,000 subjects chosen at random from the original cohort with unknown status before linkage but following use of company records, of whom there were 61% overall [(2,455+31,064)/54,509].

**Table 14:** Mortality Status at the end of follow-up (31 December 1984) in Roberts *et al*. (1989)* [9,10]

**Mortality status**	**Sudbury**	**Port Colborne**	**Total**
Dead-From company records	5,126	806	5,932
Dead-From record linkage	2,256	199	2,455
Total Dead	7,382	1,005	8,387
			
Alive-Current Employee or Pensioner	13,596	1,462	15,058
Alive-Not found to be dead	29,244	1,820	31,064
Total Alive	42,840	3,282	46,122
			
Total	50,222	4,287	54,509

* Table one in Ref. [10].

The follow-up successfully traced 925 men and found that 63 had died, of which record linkage had failed to detect 5 or 7.9% (Table 15). Of the remaining 75 men, 31 remained untraceable despite ‘herculean’ efforts by the researchers; and 44 had left the country of which partial information revealed that 13 were known to be alive and 4 dead. Roberts et al. reasoned that, if the mortality rate in the 4.4% who had left the country was similar to the unknown group as a whole, then one would expect record linkage to miss the corresponding 4.4% of deaths that occurred outside of Canada. The remaining 31 men were younger and had shorter durations of employment, on average, with likely higher rates of mobility therefore, making their trace more difficult but also rendering the assumption that their mortality rate was similar to that for the unknown group as a whole a conservative one.

**Table 15:** Comparison of record linkage and independent follow-up in Roberts *et al*. (1989)* [9,10]

**Based on record linkage**	**Based on independent follow-up**		
		
	**Dead**	**Alive**	**Totals**
Dead	58 (92.10%)	0 (0.00%)	58
Alive	5 (7.90%)	862 (100.00%)	867
Totals	63 (100.00%)	862 (100.00%)	925**

* Table two in Ref. [10]. ** 75 cases excluded: 31 not traced, 44 left country (of whom 13 known alive; 4 known dead).

With these tests, Roberts et al. reasoned that the record linkage procedure failed to detect 8% to 15% of the deaths in the unknown status group. Taking the most conservative figure, they estimated that the 2,455 deaths found by record linkage should really have been 2,455/0.85 or 2,888 deaths of which 433 would have been missed as a result. This represented a loss of 5.1% of all deaths [433/8,387] and they judged that a 95% ascertainment rate was methodologically acceptable by epidemiologic standards.

## Appendix 2

### SAS^® ^code for the logistic model calculations in Andrews and Heller (2006) [44]

options nocenter nonotes linesize = 80;

title1 'Logistic model analysis for Table three in Andrews and Heller (2006)';

data nickel;

   input r n exposure $ smoking $ cell1 cell2 cell3 cell4 cell5 cell6;

   if exposure = 'light' then exp = 0; else exp = 1;

   if smoking = 'never' then do; smo = 0; smo1 = 1; smo2 = 0; smo3 = 0; end;

   else if smoking = 'ltmed' then do; smo = 1; smo1 = 0; smo2 = 1; smo3 = 0; end;

   else do; smo = 2; smo1 = 0; smo2 = 0; smo3 = 1; end;

   smoexpint = (exp+1)*(smo+1);

   cards;

      3 100 light never 1 0 0 0 0 0

      23 160 heavy never 0 1 0 0 0 0

      33 116 light ltmed 0 0 1 0 0 0

      113 283 heavy ltmed 0 0 0 1 0 0

      16 30 light heavy 0 0 0 0 1 0

      25 49 heavy heavy 0 0 0 0 0 1

   ;

title2 'This step estimates logistic model approximations to the conditional logit model';

title3 'odds ratios in Table seven of Grimsrud *et al*. (2002) [6] [all shown in Table three]';

proc logistic nosimple; model r/n = cell2 cell3 cell4 cell5 cell6; run;

title2 'This step estimates logistic model Ni exposure odds ratios by smoking level';

proc logistic nosimple; model r/n = exp; by smo; run;

title2 'The next two steps check the statistical significance of an exposure*smoking';

title3 'interaction term in the logistic model approximation';

title4 'The first run is an additive model with the interaction term';

proc logistic nosimple; model r/n = exp smo2 smo3 smoexpint; run;

title4 'The second run is an additive model without the interaction term';

proc logistic nosimple; model r/n = exp smo2 smo3; run;

## Appendix 3

The linear logistic model refers to the logit transform of the probability of disease from exposure (P), expressed as a linear function of regression variables (x) whose values correspond to the levels of exposure to the risk factors (total nickel exposure and smoking status). The model is defined by logit P(x) = log [P/(1-P)] = α + βx. Table seven of Grimsrud *et al*. (2002) [6] shows case and control counts for 6 cells defined by 2 levels of nickel exposure, < 0.75 or ≥ -.75 mg m-3 yr, (E) and 3 levels of smoking – Never/Former, Light/Medium and Heavy (S). Those counts were estimated using a conditional logistic regression model since each case in that study was matched to one or more controls. To duplicate the counts and corresponding odds ratios would require that study's complete dataset. Since the exposure variables are not finely stratified, however, it becomes possible to approximate the Table seven results with a logistic regression model. The SAS^® ^code in Appendix 2 applies a logistic model to obtain a close approximation to the original study's odds ratio estimates (Table ten). The code also estimates the statistical significance of a nickel exposure-smoking interaction term (E*S) when added to the logistic model approximation. The models with and without E*S had χ^2 ^likelihood ratios, respectively, of 99.1857 [4 degrees of freedom (df)] and 93.4049 [3 df]. The addition of the interaction term led to an increase in the model likelihood ratio of 5.5808, a result that is statistically significant at the 5% level since Pr {χ^2 ^> 5.5808} = 0.016 [1 df]. Note: SAS^® ^software is licensed by the SAS Institute Inc., Cary, NC.

## Competing interests

Drs. Heller and Conard received financial support from Vale Inco Ltd. for the preparation of this paper. Dr. Heller also received financial support previously from Falconbridge Ltd. to conduct the underlying research in this paper. Mr. Thornhill has received no financial support.

## Authors' contributions

JGH prepared this paper and conducted its underlying research. BRC and PGT provided knowledge of the historical nickel refining processes in their respective companies; and advised on the form and content of this paper. PGT passed away on June 16, 2008 and was unable to review the final draft of this manuscript.
